# Molecular Immunology in Bacterial Vaccine Discovery

**DOI:** 10.3390/cells11233803

**Published:** 2022-11-28

**Authors:** Rita Berisio

**Affiliations:** Institute of Biostructures and Bioimaging, IBB, CNR, Via Pietri Castellino 111, I-80131 Napoli, Italy; rita.berisio@cnr.it

The global threat of antimicrobial resistance (AMR) poses a difficult challenge, as underscored by the World Health Organization (WHO), which identifies AMR as one of the three greatest threats to human health. Annual deaths due to AMR-related infections are ~700,000 and are projected to rise up to 10 million by 2050. New antibiotics are not a solution since bacteria promptly adapt and develop new resistance mechanisms. Therefore, there is a strong need to invest in vaccines against AMR infections.

This Special Issue offers an open access forum that aims to bring together a collection of nine review articles addressing multi-disciplinary approaches to unveil various aspects of vaccine development against human bacterial pathogens. Specifically, it focuses on a panel of harmful pathogens, including *Mycobacterium tuberculosis* (Mtb), AMR *Burkholderia* species, and selected ESKAPE pathogens. Among ESKAPE pathogens, a group of multidrug-resistant bacteria that are the leading cause of hospital infections globally and “escape” the biocidal action of antibiotics, we focus on Pseudomonas aeruginosa and Enterococci, both leading causes of death worldwide.

Enterococci are the second most common Gram-positive pathogen responsible for nosocomial infections. Due to the limited number of new antibiotics that reach the medical practice and the resistance of enterococci to the current antibiotic options, passive and active immunotherapies have emerged as a potential prevention and/or treatment strategy against this opportunistic pathogen. Kalfopoulou et al. explore the pathogenicity of these bacteria and their interaction with the host immune response and provide an overview of the capsular polysaccharides and surface-associated proteins that have been described as potential antigens in anti-enterococcal vaccine formulations [1]. Among Gram-positive pathogens, Mtb is a leading cause of death worldwide. The WHO “End TB Strategy” has defined goals and targets for tuberculosis prevention, care, and control to end the global tuberculosis endemic. The emergence of drug resistance and the relative dreadful consequences in treatment outcomes have led to increased awareness of immunization against Mtb. However, the proven limited efficacy of Bacillus Calmette–Guérin (BCG), the only licensed vaccine against Mtb, has highlighted the need for alternative vaccines. BCG vaccine induces some level of protection against Mtb while stimulating trained immunity that correlates with lower mortality and increased protection against unrelated pathogens. In this Issue, Pasco et al. explore BCG-induced trained immunity, including the required pathways to establish this phenotype [2]. Additionally, they discuss potential methods to improve BCG-trained immunity effects through alternative vaccine-delivery and formulation methods.

Generally, vaccines are classified into live attenuated whole-cell vaccines, inactivated whole-cell vaccines, viral-vectored vaccines, and protein subunit vaccines. Subunit vaccines are promising candidates since they can overcome safety concerns and optimize antigen targeting. They contain purified parts of the pathogen (either a protein or a polysaccharide) that are antigenic and elicit a protective immune response. Nevertheless, these vaccines need to be engineered to enhance their immunogenicity, decrease the needed antigen dose, ensure targeted delivery, and optimize antigen delivery and interaction with the immune cells. A strong contribution to vaccine improvement is given by the structural vaccinology approach and the use of adjuvants in their formulation [3]. Kramarska et al. discuss the potential of the PE_PGRS protein PE_PGRS33 as a surface antigen against Mtb and its potential use for developing new vaccines to foster a humoral response against Mtb [4]. Indeed, PE_PGRS33 interaction with Toll-like receptor 2 (TLR2) promotes the secretion of inflammatory chemokines and cytokines, which are key in the immunopathogenesis of tuberculosis [4]. This study, corroborated by successive studies [5], suggested that the PGRS domain of PE_PGRS33 exposes PGII sandwich domains on the outer surface, which are involved in the interactions with the host receptors [4]. As such, they are promising targets for a vaccination strategy aimed at inducing a humoral response. In another review, Bellini et al. focus on protein- and peptide-based subunit vaccines, examining the advantages and drawbacks of using this design approach [6]. Additionally, they explore the features of subunit vaccine candidates currently in pre-clinical and clinical evaluation and the adjuvanted delivery systems used [6]. A specific focus on adjuvants is given by Franco et al., who propose that it is advantageous to consider a wider array of immune parameters to better understand the role of adjuvants in TB immunity and establish correlates of protection [7]. Indeed, most research lines in the field of TB vaccination focus on the T-helper 1 (Th1) type of response; polyfunctional CD4+ cells simultaneously expressing IFN-γ, TNF-α, and IL-2 cytokines; and Th17 responses [7].

Infections due to Gram-negative pathogens pose a significant treatment challenge because of substantial multidrug resistance that is acquired and spread throughout the bacterial population. *Burkholderia* spp. are Gram-negative intrinsically resistant bacteria responsible for environmental and nosocomial infections. The *Burkholderia cepacia* complex (Bcc) are respiratory pathogens that primarily infect immunocompromised and cystic fibrosis patients and are acquired through contaminated products and equipment or via patient-to-patient transmission. The *Burkholderia pseudomallei* complex causes percutaneous-wound, cardiovascular, and respiratory infections. Although commercial vaccines against *Burkholderia* infections are still unavailable, substantial progress has been made in recent years in developing vaccines against *B. pseudomallei* and *B. mallei*. In this Issue, Wang et al. critically discuss the current advances in vaccine development against *B. mallei*, *B. pseudomallei*, and Bcc [8]. Additionally, Grund et al. discuss *Burkholderia* vaccine candidates derived from outer membrane proteins, OmpA, OmpW, Omp85, and Bucl8, encompassing their structures, conservation, and vaccine formulation [9].

Another Gram-negative bacterium, *Pseudomonas aeruginosa*, is a leading cause of chronic respiratory infections in people with cystic fibrosis, bronchiectasisor chronic obstructive pulmonary disease (COPD), and acute infections in immunocompromised individuals. The adaptability of this opportunistic pathogen has hampered the development of antimicrobial therapies, and consequently, it remains a major threat to public health. Due to its antimicrobial resistance, vaccines represent the sole valid alternative strategy to tackle the pathogen. However, although many advances have been made in this field in understanding the host immune response and the biology of *P. aeruginosa* over 50 years, no vaccine has yet been licensed. Sainz-Mejías et al. review the current understanding of the complexities of *P. aeruginosa*–host interactions and their implication in vaccine design [10]. Emphasis is given to understanding the current state of *Pseudomonal* vaccine development and the importance of incorporating appropriate adjuvants in the protective antigen to yield optimal protection [10].

From the host perspective, the sialic-acid-binding immunoglobulin-type of lectins (Siglecs) are receptors that recognize sialic-acid-containing glycans. In most cases, Siglecs are expressed on immune cells and play a critical role in regulating immune cell signaling. Over the years, it has been shown that the sialic-acid–Siglec axis participates in immunological homeostasis and that any imbalance can trigger different pathologies, such as autoimmune diseases or cancer. Therefore, various therapeutics have been developed that bind to Siglecs, either based on antibodies or smaller molecules. Lenza et al. provide a description of glycan-based molecules and antibody-based therapies that have been designed to therapeutically target Siglecs [11].

Taken together, data reported in this Special Issue aim to address multi-disciplinary approaches to understanding and challenging multi-resistant bacteria through vaccine development.

## Data Availability

Not applicable.
